# On the Feasibility of a pMDI-Reduced Production of Wood Fiber Insulation Boards by Means of Kraft Lignin and Ligneous Canola Hulls

**DOI:** 10.3390/polym13071088

**Published:** 2021-03-30

**Authors:** Kolja Ostendorf, Christian Ahrens, Arne Beulshausen, Jean Lawrence Tene Tayo, Markus Euring

**Affiliations:** Department of Forest Botany and Tree Physiology, Georg-August University Goettingen, 37073 Göttingen, Germany; kostend@gwdg.de (K.O.); christian.ahrens@stud.uni-goettingen.de (C.A.); arne.beulshausen@stud.uni-goettingen.de (A.B.); j.tenetayo@stud.uni-goettingen.de (J.L.T.T.)

**Keywords:** insulation, lignin, canola, propylene carbonate, physical-mechanical properties, nitrogen content, equilibrium moisture content, hot-air/hot-steam-process

## Abstract

The thermal insulation of buildings using wood fiber insulation boards (WFIBs) constitutes a positive contribution towards climate change. Thereby, the bonding of wood fibers using mainly petrochemical-based resins such as polymeric diphenylmethane diisocyanate (pMDI) is an important measure to meet required board properties. Still there is a need to reduce or partial substitute the amount of these kinds of resins in favor of a greener product. This study therefore focusses on the feasibility of reducing the amount of pMDI by 50% through the addition of 1% BioPiva 395 or Indulin as two types of softwood Kraft-Lignin and lignin rich canola hulls together with propylene carbonate as a diluent. A panel density of 160 kg/m^3^ and a thickness of 40 mm was aimed. The curing of these modified pMDI was investigated by using two types of techniques: hot-steam (HS) and innovative hot-air/hot-steam-process (HA/HS). The WFIBs were then tested on their physical-mechanical properties. The equilibrium moisture content (EMC) was determined at two different climates. An exemplary investigation of thermal conductivity was conducted as well. The WFIBs did undergo a further chemically based analysis towards extractives content and elemental (C, N) composition. The results show that it is feasible to produce WFIBs with lower quantities of pMDI resin and added lignin with enhanced physical-mechanical board properties, which were lacking no disadvantages towards thermal conductivity or behavior towards moisture, especially when cured via HA/HS-process.

## 1. Introduction

The use of wood fiber insulation boards (WFIBs) is an illustrative example for the positive contribution towards climate change. First, it stores up to 50% of carbon dioxide, which is accumulated through process of photosynthesis [1]. The storage is thereby ensured for roughly 60 years due to the durable products life-cycle [2]. Second, by proper insulation of buildings, up to 72.2% of CO_2_-emission can be saved [3]. Third, considering own CO_2_ emission of common insulating material [3], a substitution of these kind of products by wood products can yield further 1.1 t CO_2_ savings for every cubic meter of wood [2].

The bonding of wood is one of the most important measures since wood adhesives account for 65% of all available adhesives used worldwide [4]. Therefore, the type of binder used for the production for certain types of wood products constitutes an important aspect, depending on the required board properties [5]. Since the majority of common adhesives are based on fossil resources and furthermore bearing problems concerning safety and health, there is a big effort towards the utilization of natural adhesives [6]. So far, only marginal efforts have been made to investigate natural adhesives for the production of wood fiber insulation boards to produce more natural and sustainable products. Examples in this context are enzymatic activation of wood fibers [7,8,9], protein adhesive based on blood albumin [10] or an adhesive based on crude glycerol and citric acid [11].

Although, natural wood adhesives are subject to research all over the world and over decades, there is a lack of broad availability, well-studied reliance of their gluing performance and uncertain pricing when it comes to up-scaling from laboratory to industrial scale [6,12,13]. Before it is fully feasible to substitute mainstream oil-based resins with natural based alternatives, it is possible to minimize the amount of used resins by means of appropriate gluing technologies or partially substitute non-renewable with renewable resources.

Lignin is one of the most abundant natural resources in the world, accounting for 30% of the quantitative most relevant re-growing resources [14,15,16]. It is formed by an enzyme-mediated dehydrogenative polymerization of three different types of phenylpropanoid units (monolignols) to an amorphous, polyphenolic biopolymer [17]. It is an indispensable reinforcing component in all vascular plants, especially trees, by building a compound structure together with cellulose and hemicellulose which is elementary for the evolution, stability and vast vertical growth of terrestrial plants [15,16]. Nowadays, it constitutes a by-product from paper pulping. Depending on the processes behind, the yield is either a high-purity Kraft-Lignin from sulfate-process or a lower purity lignosulfonate from sulfite process. Due to its high calorific value (23 GJ t-J), most (98%) of the recovered lignin is used as combustible for pulping process. Only 2% is used in other application areas showing a high unexploited utilization potential [16,18]. Due to its chemical structure with many hydroxyl (phenolic) groups, polymeric diphenylmethane diisocyanate (pMDI) shows a high affinity towards lignin [19,20].

Worldwide, rapeseed or canola is the second largest oilseed crop after the soybean [21]. After pressing canola oil from the oilseeds, press cake is a first protein-rich by-product which can be used for feeding livestock or for further protein isolation [22]. When isolating the proteins from canola press cake, hull or hull-fragments are a further by-product, accounting for 3% of the total press cake mass [23]. The use of canola hulls is currently studied, inter alia as biosorbent for heavy metals [24], for removing dye [25] or as natural antioxidant [26].

In this context, studies were carried out investigating lignin [27,28] as natural phenolic source for phenol-formaldehyde-resins (PF). Mansouri et al. [29,30] studied a mixture of glyoxalated and hydroxymethylated lignin-solutions with PF-resin and polymeric diphenylmethane diisocyanate (pMDI) which can save amounts of these costly resins. Kirsch [7] and Kirsch et al. [9] showed a possible reduction of pMDI quantities by enzymatic pre-treatment of wood fibers with Laccase-Mediator-Systems (LMS) for the production of wood fiber insulation boards.

Furthermore, there are actual studies, utilizing natural resources as lignosulfonate as well as tannin [31] together with pMDI as crosslinking agent. Another approach is to use lignin containing cellulose nanofibrils to enhance the performance of pMDI [32] or rather partially substitute pMDI [33]. Another cutting-edge research was carried out by Barzegar et al. [34] investigating canola and soy flour as additives to pMDI for enhanced crosslinking with protein functional groups for the manufacture of plywood. However, the pre-cure of pMDI with the moisture content and hydroxyl groups of these natural polymers before application was considered to be problematic and requires a separated application on wood particles or emulsifiable pMDI [31,33].

Thompson et al. [35] patented the invention for a possible reduction of pMDI amounts together with enhanced resin distribution and avoidance of pre-curing by adding hydrophilic diluents to it. Since pMDI constitutes the predominant binder for the production of WFIB [36], this constitutes a promising concept. Therefore, this study combines these different approaches to develop a modified pMDI-resin, by mixing it with propylene carbonate as diluent (1:1), two types of softwood kraft lignin and lignin-rich canola hulls. This is designed to improve the physical-mechanical properties of the wood fiber insulation board and to save significant amounts (50%) of pMDI. The target panel density and thickness of WFIB is 160 kg/m^3^ and 40 mm. The resin cure is realized by using two different techniques: first by using hot-steam and, second, by using innovative hot-air/hot-steam-process (HA/HS) invented by Euring and Kharazipour [37].

## 2. Materials and Methods

### 2.1. Materials

#### 2.1.1. Canola hull (CH)

Canola hull (CH) was recovered as by-product from protein isolation. The original canola expeller (non-extracted and not defatted) was provided by Kleeschulte GmbH and Co. KG (Büren, Germany). Before application, the CH were vacuum dried at 100 °C to moisture content of 1–2% and pulverized using a speed rotor mill (Fritsch GmbH, Idar-Oberstein, Germany).

#### 2.1.2. Kraft Lignin

BioPiva 395 (BP) was provided by UPM Biochemicals (Helsinki, Finland) and constituted a kraft softwood lignin with 80–98 percent by weight (wt.%) and a pH between 2.5 to 4.5. The BP was vacuum-dried to moisture content ≤2% and sieved to fractions <200 µm to screen out agglomerations.

Indulin AT (IN) is a purified kraft pine lignin with 93 wt.% and a pH of 6.5, provided by Ingevity (North Charleston, SC, USA), formerly Westvaco. The ready-to-use IN was vacuum-dried to moisture content of ≤2%.

#### 2.1.3. Wood Fibers

Thermo mechanical pulp (TMP) fibers of Norway spruce (*Picea abies* Karst.) and silver fir (*Abies alba* Mill.) at a mixing ratio of 80 to 20% were provided by GUTEX Holzfaserplattenwerk H. Henselmann GmbH and Co. KG (Waldshut-Tiengen, Germany). The moisture content varied between 10% and 13%.

#### 2.1.4. Polymeric Methylene Diphenylene Diisocyanate (pMDI)

Standard pMDI I-Bond WFI 4370 from Huntsman Corporation (Rotterdam, Netherlands), with given minimum methylene diphenyl diisocyanate (MDI) of 60% and a minimum standard of 13% polyol was used in this study.

#### 2.1.5. Propylene Carbonate (PC)

1,2-Propylene carbonate (C_4_H_6_O_3_) with 99.5% purity and a molecular weight of 102.9 g/mol obtained from Acros Organics/Thermo Fisher Scientific (Waltham, MA, USA) was used as diluent in this study.

### 2.2. Methods

#### 2.2.1. Modified pMDI-Resin Preparation

**Figure 1:**
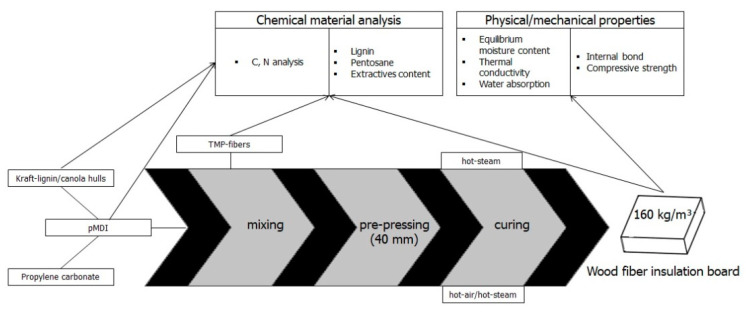
Flowchart of the experimental setup.

The pMDI (2%) was mixed together with propylene carbonate at a ratio of 1 to 1, which enhanced the viscosity on the one hand, and made it possible to add 1% of lignin or lignin rich canola hulls as additives on the other hand. In the presence of propylene carbonate, pMDI was not able to pre-cure straight forward with the reactive lignin-groups, and the water it contained [20,31,33]. Therefore, the lignin sources needed to be vacuum-dried at 100 °C for 48 h to reduce their moisture contents between 1% to 2%. The mixture was stirred for 15 min. For the purpose of comparison, 4% pMDI variants were produced which constitutes the industrial common resin load for comparable WFIBs, e.g., GUTEX Thermowall^®^ NF (GUTEX Holzfaserplattenwerk H. Henselmann GmbH and Co. KG, Waldshut-Tiengen, Germany) [38]. One of the pMDI variants was produced with propylene carbonate added to the resin and the other without propylene carbonate, this to assess the effect on the board’s properties. An overview of all produced variants is given in Table 1 and an overview of the experimental setup is given in Figure 1.

#### 2.2.2. Application on Wood Fibers and Curing

The resin-mixture was added to the wood fibers (see Figure 1) by using a pneumatic atomizer (Düsen-Schlick GmbH, Coburg, Germany) and blended with a horizontal blending unit. The moisture of the unglued fibers was between 10 to 13% to provide sufficient moistness for the reaction of pMDI with wood and its components [39]. Then, the blended fibers were loosened via airflow in the flash tube dryer. Afterwards the fibers were pre-pressed to final thickness of 40 mm and transferred to the curing unit. Two different methods were performed to cure the WFIBs. First, by adding hot-steam (HS) for about 60 s and, second, by heating up the fibers with hot air up to 120 °C within the core layer and a following injection of hot-steam for 10 s (HA/HS).

#### 2.2.3. Chemical Material Analysis

For the chemical analysis, samples of each WFIB variant were taken as well as samples of the fibers used to produce the boards (see Figure 1). Before analysis, the material was grounded using a speed rotor mill (Fritsch GmbH, Idar-Oberstein, Germany). Each sample was analyzed for its lignin content according to Klason [40], pentosane content [41] and hot as well as cold water extraction. Furthermore, an elemental (C, N) analysis was carried out. Therefore, a cured pMDI sample was analyzed as well as BP, IN and CH. The pMDI was mixed with water at a ratio of 2 to 1 to initiate a reaction, resulting in a rigid foam-like structure after a period of 48 h. The pMDI-foam was dried for 24 h and milled. The analysis was conducted via Vario Micro CubeTM elemental analyzer (Elementar Analysesysteme GmbH, Langensebold, Germany) for 5 mg of wood fiber, respectively lignin source and 2 mg for pMDI. Acetailide was used as standard.

#### 2.2.4. Thermal Conductivity and Equilibrium Moisture Content

Based on three randomly selected samples (250 mm × 250 mm), an exemplary investigation of the thermal conductivity was carried out via a two plate device (quade measurements, Langgoens, Germany) according to DIN EN 12,667 (2001). Since the board were fabricated using this industrial standard binder, no changes in thermal conductivity were expected, except for changes caused by fluctuations in specimen density. Additionally, the mass-referred equilibrium moisture content (EMC) over all variants was investigated at given standard climate conditions of 23 (±1) °C and 50% (±5%) relative humidity to evaluate possible sorption characteristic by adding different types of additives to pMDI. This process was repeated with samples conditioned at 80% (±5%) RH and 23 (±1) °C.

#### 2.2.5. Physical-Mechanical Panel Properties

The panels were tested on the following physical-mechanical properties: internal bond strength (IB), compressive strength (CS) and short-term water absorption (ST-WA). The internal bond strength (IB) was measured according to DIN EN 1607 (2013). WFIB samples (50 mm × 50 mm) were glued between two rigid wooden yokes and subsequently fixed and tested with a universal testing device (Zwick Roell, Ulm, Germany). The compressive strength (CS) was done in conformity to DIN EN 826 (2013) by applying a compressive force perpendicular to the panel plane (100 mm × 100 mm). The short-term water absorption (ST-WA) according to DIN EN 1609 (2013) was carried out by partially immersing WFIB specimen (200 mm × 200 mm) in 10 mm water for 24 h (see Figure 1).

A one-way ANOVA analysis was performed to statistically validate the results. The H_0_ hypothesis (no differences between means) was rejected if the *p*-value was less than 0.5.

## 3. Results and Discussion

### 3.1. Chemical Material Analysis

The insoluble lignin content showed strong fluctuations from 25–31% (not shown) over all variants whereas the soluble lignin content varied between 0.2–0.26% (not shown). The addition of BP, IN or CH had no measurable influence on the lignin content. According to Fengel and Wegener [15], spruce wood contains up to 27.3% lignin and silver fir up to 28.9%. This led to the conclusion that Klason is not the method of choice to analyze slight changes in lignin content by adding small amount (1%) of lignin or ligneous material. Regarding the pentosane analysis, contents of 2.4–3.1% were yielded (data not shown). Fengel and Wegener [15] published a pentosane content of 11.5% and 8.3% for silver fir and Norway spruce which are considerably higher than results obtained in this study. The major differences within the chemical material analysis were observed for cold and hot water extractions and are shown in Table 2.

For the cold water extraction, the dissolved extracts varied between 3.7 and 5.3% and their pH value was in an acidic range between 4.08 and 4.61. In relevant literature, the pH of spruce wood for cold water is 4.9 and for hot water 4.6 [15], so basically in a similar range but with a tendency to a more acidic range. The pH is not suspected to influence the performance of pMDI curing reaction [42]. Herzog [43] found a comparable amount of 4.46% for cold water extraction when investigating the same fiber mix (another batch).

The hot water extracts were higher, especially whenever propylene carbonate was involved. Due to its decomposition temperature of >241 °C [44], the substance must remain within the panel and is most likely to be eluted by hot water. Fengel and Wegener [15] reported an amount of 2% for hot water extractives, which is much lower than the results obtained in this study, even for native fibers. For the same fiber mixture, Herzog [43] found 4.7% hot water extractives. The addition of propylene carbonate, BP, IN or CR showed no explicit changes in pH of hot water extracts. Although, propylene carbonate had a neutral pH of 7 as well as IN with pH of 6.5. BP (pH 2–3) and CH (pH 4.6) are both acidic.

For a better understanding about the composition of the CH material, a comprehensive chemical analysis was carried out (Table 3). For comparison, an overview of the dry mass composition of canola hull is summarized in Natsch [45]. It shows an oil content of 14%, a protein content of 13%, a lignin content of 29–35%, and other components such as 6% phenolic substances, 1–3% sugar and up to 24% of polysaccharides. The lignin content yielded in this study (19.2%) is at least 10% lower. The oil content, detectable via petrol ether extraction, is 2% lower (12.7%) but the protein content is therefore doubled (see also Section 3.1.1). The measured 5.8% of acetone extraction accounts for sugar and phenolic substances and is mostly congruent with the pendant values in Natsch [45]. It can be assumed, that the lower lignin content comes at the expense of a higher protein content due to residual cotyledon tissue. The phenolic substances such as tannins might serve as additional reactants for pMDI. Since both, tannin and lignin, are tightly bounded within the hull structure [46] there might be a lack of accessibility for the reaction with pMDI when compared to the isolated forms of kraft lignins. The residual oil in CH might provide additional hydrophobicity of WFIBs when it comes to contact with water.

#### 3.1.1. Elemental Analysis

The results for the elemental CN analysis are shown in Table 4. Native GUTEX wood fibers had a nitrogen content 0.07%, which is almost consistent with results of Keller and Nussbaumer [47] of 0.065% for spruce and 0.06% for silver fir. The amount of 0.05% for BP kraft softwood lignin was slightly lower especially in relation to the high amount of 1.31% for IN pine kraft lignin. A study of Janshekar et al. [48] showed a similar N-content for Indulin AT pine wood kraft lignin of 1.51%. The CH showed an N-content of 4.83% corresponding to a crude protein content (N × 6.25) of 30.2% which is higher than 13% showed in Natsch [45]. This led to the assumption that the used canola hull powder is impure and contains residual protein rich seed tissue which can be confirmed by results obtained from chemical analysis (see Table 2).

The pMDI specimen showed highest nitrogen content of 9.62% as well as carbon content (69.43%) within this measurement. Köhler [49] showed a N-content of 11% for pMDI and a C-content of 72% which are similar to the results obtained in this study. When it comes to the analysis of pMDI bonded fibers there is an increase in nitrogen content from 0.07% to 0.26% for 2% pMDI added to the fibers and slightly higher 0.28% for 4% pMDI. The nitrogen content of wood panels is a measure of their resin content [50]. Naturally, the amount should be doubled, when using twice the amount of resin. Thus, it can be concluded that there is rather too much pMDI or too less pMDI on the measured samples which might be due to inconsistent spreading and wetting of the resin. By adding propylene carbonate, the N-Content was doubled from 0.21% to 0.41% when doubling the amount of pMDI. This suggests an enhanced wetting of wood fibers in comparison to samples without PC. The other variants with biopolymers as additives showed a comparable increase of nitrogen content when doubling the amount of pMDI except for variants with BP (2|2|1-BP and 4|4|1-BP). When adding canola hulls to the resin mixture, the nitrogen content slightly increased due to the nitrogen rich raw material. The carbon content of the wood fibers throughout all variants was about 50% despite the addition of higher carbon-containing material such as pMDI (69.43%) and BP (64.64%) or IN (61.92%). Köhler [49] observed also no increase in carbon content for pMDI-bonded particleboards.

### 3.2. Thermal Conductivity

The exemplary investigation of the thermal conductivity for randomly chosen WFIB specimens is shown in Table 5. It is apparent that thermal conductivity increased from 0.039 to 0.042 W/(m × K) with increasing specimen densities from 150 to 168 kg/m^3^ but the increase was not significantly different (*p* > 0.5). The obtained values are congruent with literature references. Wieland et al. [51] measured a thermal conductivity of 0.039 W/(m × K) for a panel with a density of about 149 kg/m^3^. Herzog [43] measured thermal conductivities between 0.40 and 0.41 W/(m × K) for 160 kg/m^3^ panels. The thermal conductivity is thereby mostly dependent on panel density [36,43,52]. With increasing densities, the porosity and pore size decreased and thus the heat transfer [53]. The density fluctuations are caused by inhomogeneous fiber spreading in laboratory scale production process. Other impact factors are moisture content or used type and quality of fiber [51,53]. Due to the identical climatic conditioning, an influence of panel moisture could be excluded.

#### Equilibrium Moisture Content (EMC)

**Figure 2:**
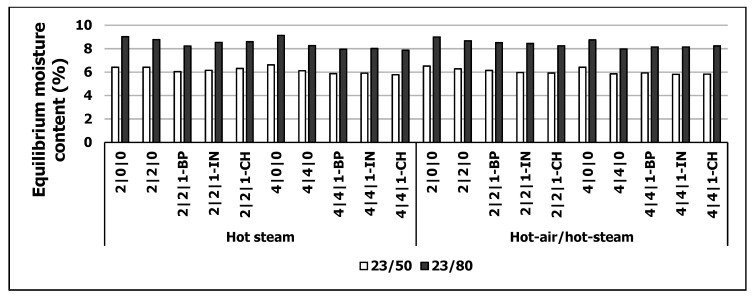
Equilibrium moisture content (%) of wood fiber insulation boards using hot steam (HS) and hot-air/hot-steam (HA/HS) for curing at two different climate conditions: 23 °C, 50% RH (23/50) and 23 °C, 80% RH (23/80).

The EMC of the WFIB samples after 48 h storage under two different climates (23 °C, 50% RH/23 °C, 80% RH) are shown in Figure 2.

At standard climate, the EMC of the produced WFIBs varied between 5.8% (4|4|1-CH, HA/HS) and 6.6% (4|0|0, HA/HS). There was no significant difference in EMC for both curing methods (*p* > 0.5). Though, there is a small tendency towards higher EMCs for HS variants. At the higher humidity of 80%, the moisture content (MC) increased from 2% over all variants up to 9% (2|0|0, HS). Again, there was no clear tendency in EMC changes between the two curing methods. Brombacher [54] found no significant differences in EMC for WFIB products produced via dry or wet process and concludes that the sorption of WFIBs is mainly influenced by the fiber itself and not the processing technique to a fiber composite.

In general, the MC was slightly lowered with increasing amount of pMDI. A slight decrease in moisture content with increasing amount of pMDI was observed in Hunt et al. [55] for low density fiberboard which might be due to higher amount of pMDI-blocked OH-groups. The addition of propylene carbonate to pMDI resulted in a slightly lower moisture content which might be due to the enhanced spreading of the resin and, thus, a higher degree of blocked OH-groups on a larger surface area.

By adding BP, IN or CH to the resin, there was a decrease in moisture content in comparison to the references (2|0|0 or 4|0|0) under both climates. However, the variants with added BP, IN or CH were approximately at the same level as 2|2|0 and 4|4|0 variants. In particular, a negative impact on water absorption from surrounding environment for the used additives could be excluded. For example, Kraft lignin is considered as hydrophobic [56], so an additional effect on water absorption can be ruled out. Interestingly, the 2% pMDI variants with added PC and lignin showed a superior behavior towards moisture absorption than using twice the amount of pMDI. These results imply that modified pMDI is well spread onto fiber surface.

A negative impact of curing method regarding MC can be excluded and, thus, an impact on physical-mechanical board properties or thermal conductivity [51,53,54].

### 3.3. Physical-Mechanical Panel Properties

#### 3.3.1. Internal Bond Strength (IB)

**Figure 3:**
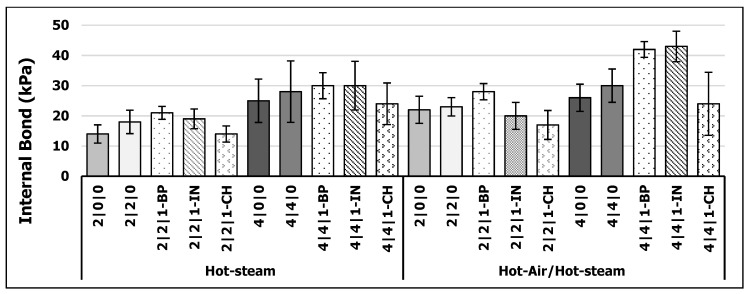
Internal bond strength (IB) of WFIBs (kPa) cured via hot-steam and hot-air/hot-steam process (*n* = 30).

The results (*p* < 0.5) for IB are shown in Figure 3. There was an obvious increase in IB when doubling the amount of pMDI from 2% to 4%. The results for merely 2% pMDI bonded WFIBs were satisfactory compared to the industrial 4% pMDI bonded WFIBs board from GUTEX company (160 kg/m^3^, 40 mm) which required a minimum IB of 10 kPa [38]. It is apparent that, by adding PC to the pMDI, the IB increased independently of the curing method. This is due to enhanced resin spreading during resination and therefore enhanced wetting of wood fibers [35]. The results of the elemental analysis suggested a more homogenous resin spreading when adding PC to pMDI.

**Figure 4:**
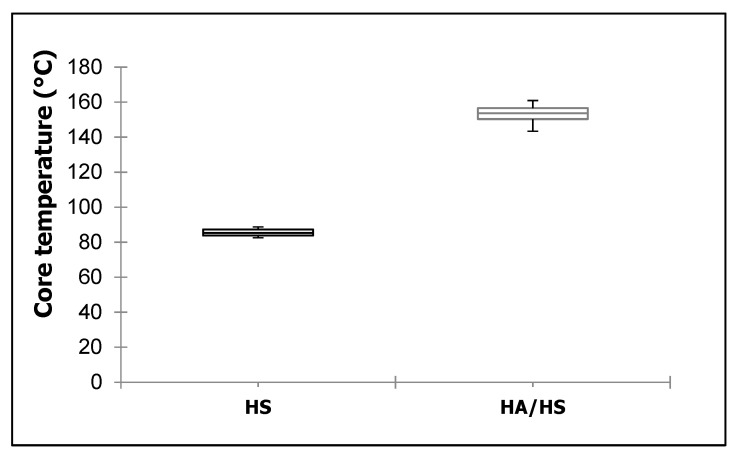
Maximum temperatures within the different curing processes: hot-steam (HS) and hot-air/hot-steam (HA/HS). For each process, the results of 30 individually produced boards are displayed (*n* = 30).

The HA/HS treatment had a beneficial effect on IB values in comparison to HS variants. pMDI is known to form polyurea, biuret/polyuret and urtehan linkages to the wood within the bond line [20,57,58]. pMDI tends to react with moisture content of wood in the first place to form amines which can further react to polyurea structure [57]. When the moisture content decreases with increasing temperatures, for example when heating up with hot air, the pMDI is eager to react with hydroxyl groups of wood in the second place to form covalent urethane structures [20,57,58]. As a side reaction, the pMDI can also react with previously formed polyurea linkages to biuret structure [20]. Therefore, temperature plays an important role in terms of accelerating the curing reaction [58]. Bao et al. [57] found, that temperature of 140 °C promotes the reaction between pMDI and wood by forming biuret bonds and thus an enhanced crosslinking. By taking a look on the maximum temperatures within the two curing processes (see Figure 4), the HA/HS curing method reached an average temperature of 153.2 °C. The HS process, however, showed merely 92.4 °C. Thus, it can be assumed that the higher curing temperature provides an accelerated pMDI reaction to wood as mentioned above. Besides moisture content and process parameters such as temperature, the particle characteristics (e.g., fibers, strands) and wood species are also of importance for curing pMDI [59].

By adding 1% of BP or IN to the resin, there is an increase in IB, especially for HA/HS variants. It may be assumed, that especially the types of kraft lignin serve as additional crosslinking agent by providing phenolic groups for the reaction with pMDI [20,57]. In comparison, the addition of CH resulted in lower IB. This might be due to its impurity and comparatively low lignin content (see Table 3), and a possible lack of accessibility as discussed above [46]. The IB constitutes a measure for the gluing quality; therefore, it is possible to derive new findings about the performance of the different variants. That there is a greater increase in IB for 4% pMDI variants under addition of BP and IN ought to be due to the higher amount of pMDI as reactant. However, the IB for 2% variants with BP (2|2|1-BP) is able to compete with 4% variants (4|0|0), in particular when using HA/HS method. Regarding IB, the pMDI-reduced production of WFIBs is possible, especially when using BP as additive together with PC.

#### 3.3.2. Compressive Strength (CS)

**Figure 5:**
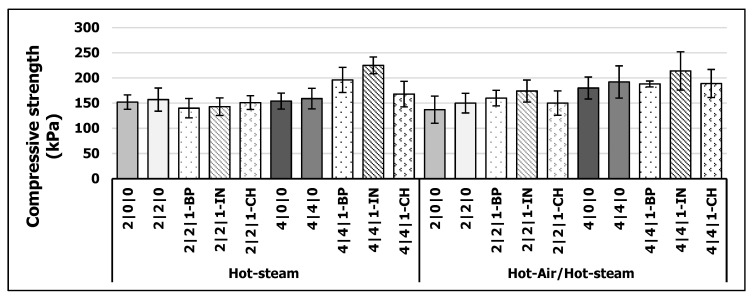
Compressive strength (CS) of WFIBs (kPa) cured via hot-steam and hot-air/hot-steam process (*n* = 24).

The results (*p* < 0.5) for CS are displayed in Figure 5. There was a slight increase in CS when diluting pMDI with PC, which was especially more pronounced for HA/HS variants. For HS variants, there was no increase in CS when doubling the amount of pMDI. In contrast, when using HA/HS curing, there is an increase in CS when using twice the amount of pMDI. This might be due to enhanced reaction by curing with higher temperatures as discussed for IB. Furthermore, enhanced mobility of the pMDI polymer with increasing temperatures might promote the spreading within the fiber mat as well as curing behavior [60]. The results of 2% pMDI bonded WFIBs are satisfactory compared to an industrial 4% pMDI bonded WFIBs board from GUTEX company (160 kg/m^3^, 40 mm) which requires a minimum CS of 100 kPa [38].

The CS is mainly influenced by panel density, bulk density and fiber size [61]. Thus, there is no such change in CS with increasing pMDI-content and between the two curing methods when comparing to IB results. To analyze a possible influence of bulk density and fiber size, the native fibers were additionally investigated on these two properties as shown in Table 6.

The bulk density of the used TMP fibers was 16.4 kg/m^3^. The fiber length weighted by area, measured via FibreShape, showed 0.28 mm for 10% percentile, a median value of 1.84 mm and 4.38 mm for 90% percentile based on total volume. The fiber length was basically dependent on pulping conditions and influences the strength properties of a WFIB. Fengel and Wegener [15] listed a fiber (tracheid) length of 1.7 to 3.7 mm for Norway spruce and 3.4–4.6 mm for silver fir. These figures are congruent with at least 50% of the measured data (>50% percentile). Smaller particles occur due to the refining process and partially ruptured cell walls [15]. The bulk density is equally dependent on fiber size distribution. The obtained bulk density is comparable with a result (16.2 kg/m^3^) of the same fiber mix but from another batch used [61]. The bulk density of used TMP fibers must be significantly lower than the desired panel density (160 kg/m^3^) to provide a higher compression ratio and, thus, a more solid panel [61]. The smaller the fiber size of a pulp, the higher the bulk density and vice versa [61]. It can be assumed that the used fibers are, with regard to their industrial origin and their morphology, well suited for the manufacture of WFIB.

By adding BP, IN or CR to the pMDI/PC mixture, there is an increase in CS especially for 4% pMDI variants. The more pMDI is available, the higher the possibility to react with lignin or ligneous material. In comparison to IB, the CR variants are more on same level as BP variants. With a high converted protein content of 30.6% for CH, pMDI might react with residual amino-groups to form urea [34] which can further react to biuret structure which are more rigid [57]. This may enhance CS but is not beneficial for improving IB. IN seems to be the best additive to enhance CS, independent of the curing process. This might be due to its higher nitrogen content in comparison to BP, which might improve CS in a similar manner as discussed for CH.

#### 3.3.3. Short-Term Water Absorption (ST-WA)

**Figure 6:**
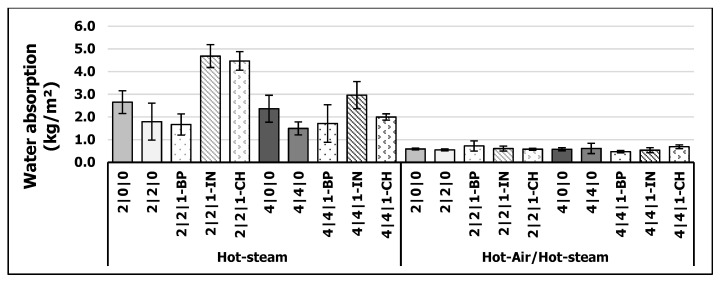
Short-term water absorption (ST-WA) of WFIBs (kg/m^2^) cured via hot-steam and hot-air/hot-steam process (*n* = 9).

The results for ST-WA are shown in Figure 6. There was an explicit decrease in ST-WA when curing with HA/HS-process. All of the variants (*p* > 0.5) showed a water uptake less than 1 kg/m^2^, which is the required specification for comparable GUTEX product [38]. This might be due to the higher amount of covalent bonds as discussed for IB (see Section 3.3.1). According to Zhou and Frazier [62] an increasing amount of covalent bond might be beneficial for strong bond line behavior and a higher resistance towards moisture. That HA/HS curing method provide pMDI bonded WFIB with improved water resistance was also observed in [8,9,10]. Kirsch et al. [9] produced WFIBs with a density of 180 kg/m^3^ and pMDI content of only 1% and measured a ST-WA < 1 kg/m^2^. Regarding HS-process, there is a decrease in ST-WA when adding PC to the pMDI. When doubling the amount of pMDI, the ST-WA naturally decreases as well. The addition of BP leads to a further decrease when curing with HS, at least for the 2% variant. However, the addition of CH and especially IN led to an increase in ST-WA for 2% and 4% pMDI variants. This observation is remarkable because the results for MC measurements showed no tendency towards a higher sorption behavior by adding IN or CH. The oil content of CH (see Table 3) has therefore no additional hydrophobic effect on ST-WA as previously presumed.

## 4. Conclusions

This study dealt with the feasibility of 50% pMDI reduced production of WFIBs by adding 1% of softwood kraft lignin or ligneous canola hulls and propylene carbonate (PC) as diluent. Thereby, two curing processes were used, the hot steam (HS) and innovative hot-air/hot-steam process (HA/HS). The results show that it is possible to reduce the amount of pMDI by 50% for both processes regarding mechanical properties. However, for HS process, the short-term water absorption (ST-WA) is much higher throughout all variants compared to HA/HS process. In general, the HA/HS process shows enhanced board properties especially regarding internal bond strength (IB) and ST-WA. It is assumed that the higher process temperature led to enhanced resin cure and a higher amount of covalent bonds. In both processes, the board properties were positively influenced, in the first place, by addition of propylene carbonate due to an enhanced resin spreading. This phenomenon could be underlined by changing nitrogen contents of the WFIBs measured via elemental analysis. In the second place, there was a further improvement when adding kraft lignin to the pMDI/PC mixture. Between the two types of kraft lignin, BioPiva 395 (BP) showed a more consistent performance compared to Indulin AT (IN) especially with regards to ST-WA. The addition of lignin rich canola hulls (CH), however, led to a decrease in internal bond strength (IB) and an increase in ST-WA. This might be due to measured impurity and an assumed lack of accessibility of the reactive components (lignin, tannin) for pMDI. There was no effect on the additives (PC, BP, IN, CH) regarding thermal conductivity or sorption behavior measured via moisture content in two different climates. Nonetheless, there is a need of further investigations regarding cure dynamics. In this context, solid state NMR or DSC studies should be performed. A short-term goal would be the reduction of propylene carbonate content in order to minimize costs and to improve economic efficiency. The next step would be an industrial establishment of HA/HS-process so that upscaling to industrial scale becomes possible.

## Figures and Tables

**Table 1:** Overview of the modified polymeric diphenylmethane diisocyanate (pMDI)-resin formulations based on absolutely dry mass of the wood fibers.

Variant	pMDI (%)	Propylene Carbonate (%)	Additive (%)		
			BioPiva 395 (BP)	Indulin AT (IN)	Canola Hulls (CH)
2|0|0	2	0	0	0	0
2|2|0	2	2	0	0	0
2|2|1-BP	2	2	1	0	0
2|2|1-IN	2	2	0	1	0
2|2|0-CH	2	2	0	0	1
4|0|0	4	0	0	0	0
4|4|0	4	4	0	0	0
4|4|1-BP	4	4	1	0	0
4|4|1-IN	4	4	0	1	0
4|4|1-CH	4	4	0	0	1

**Table 2:** Chemical analysis of WFIB specimen with respect to native TMP fibers according to pH values and extracts by cold and hot water extraction (*n* = 3).

Variant	Cold Water		Hot Water	
	pH	Extracts (%)	pH	Extracts (%)
native	4.32	4.5	4.40	4.8
2|0|0	4.59	5.3	4.59	4.6
2|2|0	4.33	5.0	4.33	8.1
2|2|1-BP	4.44	4,4	3.98	6.3
2|2|1-IN	4.40	4.3	4.45	6.4
2|2|1-CH	4.39	4.6	4.23	8.8
4|0|0	4.47	5.3	4.47	5.3
4|4|0	4.44	5.2	4.44	8.5
4|4|1-BP	4.08	4.9	4.56	8.2
4|4|1-IN	4.61	3.7	4.56	7.3
4|4|1-CH	4.43	4.2	4.88	8.4

**Table 3:** Chemical analysis of pulverized canola hulls according to lignin, pentosane and extractives content (*n* = 3).

Canola Hull Analysis			
Lignin	soluble (%)	1.2	∑
	insoluble (%)	18	19.2
Pentosane	(%)	2.5	
Cold water	extracts (%)	24.8	
Hot water	extracts (%)	25.9	
	pH	4.6	
Successive extraction	petrolether	12.7	∑
	diethylether	0.6	23.0
	acetone	3.9	
	ethanol	5.8	

**Table 4:** Results for elemental analysis regarding carbon (C) and nitrogen (N) content (%) for WFIB variants and based raw materials (*n* = 3).

	N (%)	C (%)
Native fiber	0.07 (±0.004)	49.3 (±0.13)
BioPiva395 (BP)	0.05 (±0.003)	64.6 (±0.41)
Indulin (IN)	1.31 (±0.006)	61.9 (±0.05)
Canola Hull (CH)	4.83 (±0.025)	49.7 (±0.11)
pMDI	9.62 (±0.079)	69.4 (±0.30)
2|0|0	0.26 (±0.007)	48.5 (±0.98)
4|0|0	0.28 (±0.009)	48.4 (±1.50)
2|2|0	0.21 (±0.002)	50.3 (±0.87)
4|4|0	0.41 (±0.006)	49.8 (±0.62)
2|2|1-BP	0.31 (±0.016)	48.6 (±0.60)
4|4|1-BP	0.35 (±0.017)	50.0 (±0.32)
2|2|1-IN	0.20 (±0.005)	49.2 (±0.33)
4|4|1-IN	0.37 (±0.008)	50.6 (±0.34)
2|2|1-CH	0.27 (±0.005)	49.7 (±0.11)
4|4|1-CH	0.47 (±0.007)	50.8 (±0.13)

**Table 5:** Thermal conductivity of randomly selected WFIBs according to their density.

Sample	Density	Average Density	Thermal Conductivity	Variant	Curing
	(kg/m^3^)	(kg/m^3^)	(W/(m × K))		
1.1	145.57	150.2	0.039	(2|2|1-BP)	HA/HS
1.2	154.77				
2.1	167.84	168.2	0.042	(4|4|1-IN)	HS
2.2	168.56				
3.1	157.78	156.2	0.040	(2|2|1-IN)	HA/HS
3.2	154.51				

**Table 6:** Results for bulk density (*n* = 5) and fiber length measurement via FibreShape (*n* = 2) for TMP fibers made from 80% norway spruce and 20% silver fir.

Bulk Density	Geodesic Fiber Length (mm)		
(kg/m^3^)	Percentile (Q_2_)		
	10	50	90
16.4 (±0.7)	0.28 (±0.01)	1.84 (±0.07)	4.38 (±0.04)

## Data Availability

The data presented in this study are available on request from the corresponding author.
